# Repeatability and reproducibility of MRI apparent diffusion coefficient applied on four different regions of interest for patients with axial spondyloarthritis and healthy volunteers scanned twice within a week

**DOI:** 10.1259/bjro.20200004

**Published:** 2020-12-21

**Authors:** Jakob Møllenbach Møller, Mikkel Østergaard, Henrik S Thomsen, Stine Hangaard, Inge J Sørensen, Ole Rintek Madsen, Susanne J Pedersen

**Affiliations:** 1 Department of Radiology, Herlev-Gentofte Hospital, Herlev, Denmark; 2 Department of Clinical Medicine, Faculty of Health and Medical Sciences, University of Copenhagen, Copenhagen, Denmark; 3 Copenhagen Center for Arthritis Research, Center for Rheumatology and Spine Diseases, Rigshospitalet, Glostrup, Denmark

## Abstract

**Objectives:**

The apparent diffusion coefficient (ADC) may be used as a biomarker for diagnosis and/or monitoring treatment response in patients with axial spondyloarthritis (axSpA), but this requires reliable ADC measurements. This study assessed test–retest repeatability and reproducibility of ADC measurements using four different region of interest (ROI) settings.

**Methods:**

In this prospective study, the sacroiliac joints (SIJs) of 25 patients with axSpA and 24 age- and sex-matched healthy volunteers were imaged twice at a mean interval of 6.8 days in a 1.5 T scanner using, multishot echoplanar diffusion-weighted sequences. ADCs at four ROI settings were assessed: 5 mm and 10 mm anatomic band-shaped, 15 mm linear, and 40 mm^2^ circular.

**Results:**

Intraclass correlation coefficient (ICC) assessments showed that the interstudy repeatability was good for median ADC (ADC_med_) and 95th-percentile ADC (ADC_95_) measurements in patients with axSpA (0.77–0.83 and 0.75–0.83, respectively), but poor-to-moderate in healthy subjects (0.27–0.55 and 0.13–0.37, respectively). For all ROI settings, intrareader reproducibility was excellent for ADC_med_-measurements (ICC:0.85–0.99) and moderate-to-excellent for ADC_95_ measurements (ICC:0.68–0.96). The 5 mm ROI had the least estimated bias and highest level of agreement on Bland–Altman plots. The interreader reproducibility was moderate (ICC:0.71). The 15 mm linear ROI produced significantly greater ADC_med_ and ADC_95_ measurements than all other ROI settings (*p* < 0.01–0.02), except for the circular ROI ADC_95_ measurements.

**Conclusion:**

ROI settings influence ADC measurements. Interstudy repeatability of SIJ ADC measurements is independent of ROI settings. However, the 5 mm ROI showed the least bias and random error and seems preferable.

**Advances in knowledge:**

ADC measurements are affected by ROI settings, and this should be taken into account when assessing ADC maps.

## Introduction

The chronic inflammatory disease axial spondyloarthritis (axSpA) causes severe pain and functional disability, and with time often structural bone damage and, ultimately, ankylosis.^1^ Bone marrow edema (BME), which can be detected by short tau inversion recovery (STIR) MRI sequences, is a highly sensitive but less specific indicator of inflammation.^2^ The apparent diffusion coefficient (ADC) is derived from diffusion-weighted imaging (DWI) and has been investigated as a potential biomarker of axSpA disease activity in diagnostic studies and studies monitoring responses to treatment.^3–7^ However, the assessment of ADC maps has not been standardized and several approaches have been used. Circular regions of interest (ROIs) of various sizes (70–90 mm^2^) have been used in predefined areas of the sacroiliac joints (SIJs),^3,5,7^ as have circular or polynomial ROIs positioned on areas affected by BME.^4,6,7^ One study used a linear ROI that extended from the bone marrow on one side of the joint to the other, covering both the iliac and sacral bone marrow and including the joint cavity.^8^ Using an ROI that covers the bone marrow entirely was also suggested recently.^9^ Several relevant studies have measured interobserver reproducibility,^3,6,8^ whereas few studies have measured intraobserver reproducibility.^3^ However, to our knowledge, the interstudy repeatability of SIJ measurements has not been investigated (*i.e.* repeatability between two MRI scans performed within a short period, or test–retest reliability). Therefore, we investigated variation, interstudy repeatability, and intra- and inter reader reproducibility of ADC measurements using four different types of standardized ROI settings in patients with axSpA and healthy subjects.

## Methods and materials

### Study design

This was a prospective test–retest study involving patients with axSpA and sex- and age-matched healthy volunteers. MRI images of the SIJs were acquired twice within 7 ± 2 days. Inclusion criteria for the patients were: (1) AxSpA according to the Assessment of Spondyloarthritis International Society (ASAS); (2) inflammatory back pain as judged by an expert rheumatologist. Exclusion criteria for the patients were: (1) glucocorticoid injections or initiation of/changes to oral glucocorticoid or tumor necrosis factor-inhibitor dose within 3 months prior to study start; (2) dose changes in non-steroidal anti-inflammatory drugs (NSAIDs) during the study or within 2 weeks prior to the study starting. Patients were recruited from the rheumatology clinics at Gentofte and Glostrup hospitals. Exclusion criteria for the healthy subjects were: (1) arthritis or pain in the peripheral joints or spine during the preceding 3 months. (2) Moreover, the healthy subjects were not allowed to have first- or second-degree relatives with axSpA, psoriatic or rheumatoid arthritis. Healthy subjects were recruited from the local radiology department. The study was approved by the Ethical Committee of the Capital Region of Denmark (approval no. H-3-2012-085) and all subjects provided written informed consent before any study procedures. Further details of this study, including clinical assessments, have previously been published elsewhere.^10^

### MRI technique

MRI of SIJs was performed twice in each subject using a combination of a 5-channel spine coil and a two channel flexible coil in a 1.5 T MR system (Achieva; Philips, Best, The Netherlands). The sequence settings were as follows: *T*_1_ weighted (*T*_1_W): time to repeat (TR) = 550 ms, time to echo (TE) = 14 ms, slice thickness (ST) = 4 mm, spatial resolution (SR) = 0.9 × 1.6 mm^2^; STIR: TR = 2550 mms, TE = 60 ms, time to invert (TI) = 160 ms, ST = 4 mm, SR = 1.3 × 1.6 mm^2^; and multishot echoplanar imaging DWI: TR = 2000 mms, TE = 75 ms, ST = 5 mm, SR = 2.1 × 2.1 mm^2^, *b* = 0, 50, 500, 800 s mm^−2^. ADC maps were calculated based on all b-values using vendor specific software (Intellispace v. 6, Philips, Best, The Netherlands). All sequences were obtained in the semi-coronal plane.^10^

### Anonymization

All examination results from time point 1 (MR1; *n* = 49) and time point 2 (MR2; *n* = 49) were pooled into one image database and anonymized. Further, the examinations from MR2 (*n* = 49) were re-anonymized using different numbers and added to the database to assess the intrareader variation. In total, 147 sets of SIJ data were available for image analysis.

### Image analysis

**Figure 1.**
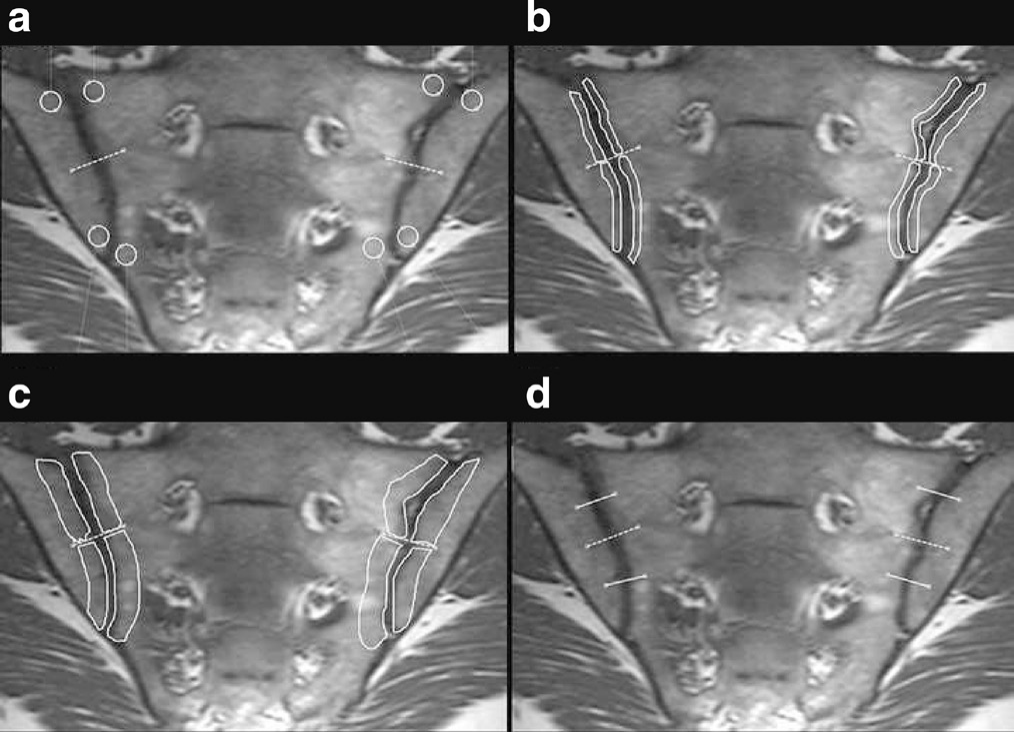
Semicoronal T1-weighted magnetic resonance image showing the four ROIs that were assessed: 40 mm^2^ circular (a); 5 mm anatomic band-shape (b); 10 mm anatomic band-shape (c); and 15 mm linear (d). ROIs, regions of interest.

Each SIJ was divided into four quadrants where four different ROI settings were tested (Figure 1): a 40 mm^2^ circular ROI located proximally and distally in the iliac and sacral bone marrow; a free hand-drawn anatomic band-shaped ROI covering the entire length of the SIJ quadrant to a perpendicular depth of 5 mm from the joint cavity; a similar 10 mm free hand-drawn anatomic band-shaped ROI; and finally, a 15 mm linear ROI positioned at the midpoint of each joint half, perpendicular to the joint cavity and covering the bone marrow in two quadrants equally. The linear ROI included the joint space between the bones, whereas the other ROIs only covered the bone marrow. Assessments were performed using 4 consecutive slices, resulting in 32 ROIs per subject for the first 3 ROIs and 16 ROIs for the 15 mm ROI. For each ROI setting, the median ADC (ADC_med_) and 95th percentile ADC (ADC_95_) values were calculated from the 32 (16) ADC measurements. The most anterior slice where more than 1 cm of the SIJ was visible was defined as the first slice. A senior radiographer with >10 years’ experience in axSpA and body DWI performed all ADC assessments. Inter-reader assessment was performed at 10 (20%) randomly chosen 5 mm ROI cases by a resident with 6 years’ experience in muscle-skeletal imaging.

All SIJ MRI examination results were evaluated for BME using the Spondyloarthritis Research Consortium of Canada (SPARCC) SIJ inflammation score (SPARCC-BME), and assessed for structural lesions (*i.e.* fat lesions, erosion, backfill, and ankylosis) according to the SPARCC SIJ structural score (SPARCC-SSS), using *T*_1_W and STIR images. These evaluations were performed by one reader with 15 years of experience in MRI of SIJs affected by axSpA.

### Statistics

Variation in disease activity between MRI1 and MRI2, measured using the SPARCC-BME and SPARCC-SS, was quantified using paired *t*-tests. Due to slight skewness of the data the ADC_med_ was used as central tendency instead of mean ADC. Absolute agreement was assessed using Bland–Altman plots. Furthermore, the mean difference between MRI1 and MRI2 (mentioned bias) was assessed using a one-sample *t*-test. The standard deviation (SD) of the differences between the MRI1 and MRI2 examination results was mentioned random error. The 95% limits of agreement were calculated as ±1.96 × SD. MRI1 and MRI2 examination results were assessed using linear regression. Interstudy repeatability (*i.e.* the variance between repeated measurements – in this case between two MRI scans) intrareader reproducibility and interreader reproducibility (*i.e.* the objectivity of the assessor(s)) were both assessed using a single measure two-way mixed intra class correlation coefficient (ICC). The ICC results were interpreted as follows: <0.5: poor, 0.51–0.75: moderate, 0.76–0.90: good, and >0.91: excellent.^11^ These assessments were performed for both ADC_med_ and ADC_95_ measurements. The four ROI settings were compared using one-way analysis of variance (ANOVA) and Tukey’s honestly significant difference *post-hoc* test. The statistical software package SPSS (v. 22.0; IBM, Armonk, NY) was used for all analyses. *p*-values below 0.05 were considered statistically significant.

## Results

### Population

A total of 25 patients with axSpA (12 females mean age 36.1 SD 9.9, 13 males mean age 41.9 SD 10.3) and 24 healthy control subjects (11 females mean age 42.6 SD 13.3, 13 males mean age 44.4 SD 7.6) participated in the study. There were no statistically significant differences in age for either females (*p* = 0.20) or males (*p* = 0.48).

### Variation in disease activity

No changes were observed between MRI1 and MRI2 examinations in patients with axSpA for the SPARCC-BME (*p* = 0.71) or for the SPARCC-SS regarding ankylosis (*p* = 0.39), erosion (*p* = 0.08), fat (*p* = 0.67), or backfill (*p* = 0.83). Similarly, no changes were observed in healthy subjects for SPARCC-BME (*p* = 1.00) or SPARCC-SS regarding fat (*p* = 0.33). No ankylosis, erosion, or backfill were observed in healthy subjects. In the axSpA group 28% had BME lesions.

### Correlations of ADC

**Figure 2.**
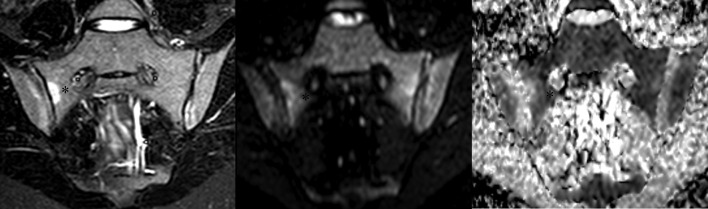
Oblique axial STIR (left), DWI (middle) and ADC (right) of the sacroiliac joints. A bone marrow edema is present in the sacral part of the right SI joint (asterisk). There is a similar finding on DWI (*b* = 50 s/mm^2^), whereas the lesion is larger on the ADC map. In the iliac part of the right SI joint a thin subchondral BME is present on STIR and DWI, while larger on the ADC map. ADC, apparent diffusion coefficient; DWI, diffusion-weighted imaging; SI, sacroiliac; STIR, short-tau inversion recovery.

There was a highly positive correlation of ADC_med_ with mean ADC (0.98 and 0.94; both *p* < 0.01) for axSpA patients and healthy volunteers, respectively. Both ADC_med_ and ADC_95_ were positively correlated with SPARCC-BME score in axSpA patients (Figure 2) but no correlation of structural scores with any ADC was revealed.

### Results for the four different ROI settings

Results for the four different ROI settings in patients with axSpA and healthy subjects are shown in Table 1. For all ROI settings, both ADC_med_ and ADC_95_ measurements in patients with axSpA and healthy subjects were tested using one-sample *t*-tests, and the differences between MRI1 and MRI2 examinations were not significantly different from 0, indicating that there was no systematic bias.

**Table 1.** Median and 95th percentile ADC measurements from four different regions of interest at two MRI examinations (MRI1 and MRI2)

	5 mm band-shaped ROI	10 mm band-shaped ROI	15 mm linear ROI	40 mm^2^ circular ROI
ADC_med,_ axSpA (*n* = 25)				
MRI1 ADC_med_, mean (SD)	644.4 (164.2)	722.8 (216.3)	797.2 (251.8)	676.8 (182.8)
MRI2 ADC_med_, mean (SD)	649.4 (186.4)	737.5 (290.7)	832.3 (295.4)	716.3 (262.2)
Difference (SD)	5.0 (113.3)	13.5 (149.1)	24.8 (192.1)	42.9 (153.3)
Interstudy repeatability (95% CI)	0.79 (0.58–0.90)	0.83 (0.65–0.92)	0.82 (0.62–0.92	0.77 (0.54–0.90)
Intrareader reproducibility (95% CI)	0.92 (0.82–0.96)	0.99 (0.97–1.00)	0.95 (0.88–0.98)	0.98 (0.95–0.99)
ADC_med_, healthy controls (*n* = 24)				
MRI1 ADC_med_, mean (SD)	635.2 (119.6)	669.6 (145.1)	822.2 (195.2)	619.3 (154.5)
MRI2 ADC_med_, mean (SD)	641.5 (96.5)	685.0 (104.8)	825.6 (176.8)	643.3 (112.2)
Difference (SD)	19.8 (127.6)	21.3 (140.9)	31.3 (288.8)	30.7 (140.3)
Interstudy repeatability (95% CI)	0.27 (–0.17–0.61)	0.37 (–0.04–0.68)	0.55 (0.18–0.78)	0.45 (0.06–0.72)
Intrareader reproducibility (95% CI)	0.95 (0.88–0.98)	0.91 (0.80–0.96)	0.85 (0.69–0.93)	0.92 (0.83–0.97)
ADC_95,_ axSpA (*n* = 25)				
MRI1 ADC_95_, mean (SD)	1126.5 (352.8)	1123.9 (333.2)	1288.0 (342.5)	1203.0 (332.3)
MRI2 ADC_95_, mean (SD)	1209.9 (363.5)	1172.4 (353.1)	1301.7 (316.4)	1234.4 (336.1)
Difference (SD)	83.5 (284.3)	46.4 (184.2)	7.5 (220.9)	32.7 (237.9)
Interstudy repeatability (95% CI)	0.78 (0.56–0.90)	0.83 (0.65–0.92)	0.78 (0.56–0.90)	0.75 (0.51–0.89)
Intrareader reproducibility (95% CI)	0.73 (0.47–0.87)	0.96 (0.91–0.98)	0.83 (0.65–0.92)	0.91 (0.80–0.96)
ADC_95,_ healthy controls (*n* = 24)				
MRI1 ADC_95_, mean (SD)	1058.7 (229.8)	1042.4 (279.0)	1269.3 (231.3)	1072.2 (277.2)
MRI2 ADC_95_, mean (SD)	1056.0 (177.2)	1064.1 (199.4)	1290.7 (261.3)	1106.2 (212.9)
Difference (SD)	14.4 (269.4)	32.6 (293.6)	35.4 (295.4)	46.9 (275.5)
Interstudy repeatability (95% CI)	0.13 (–0.30–0.51)	0.26 (–0.16–0.60)	0.27 (–0.15–0.61)	0.37 (–0.04–0.67)
Intrareader reproducibility (95% CI)	0.68 (0.39–0.85)	0.87 (0.71–0.94)	0.71 (0.44–0.87)	0.85 (0.69–0.93)

ADC_95_, 95th percentile apparent diffusion coefficient; ADC_med_, median apparent diffusion coefficient; 95% CI, 95% confidence interval; ICC, intraclass correlation coefficient; MRI1, first MRI examination; MRI2, second MRI examination; ROI, region of interest; SD, standard deviation; axSpA, axial spondyloarthritis.

Differences between the measurements (*i.e.* bias), random error (*i.e.* standard deviation of the differences), the interstudy repeatability, and the interstudy reproducibility are also shown.

**Figure 3.**
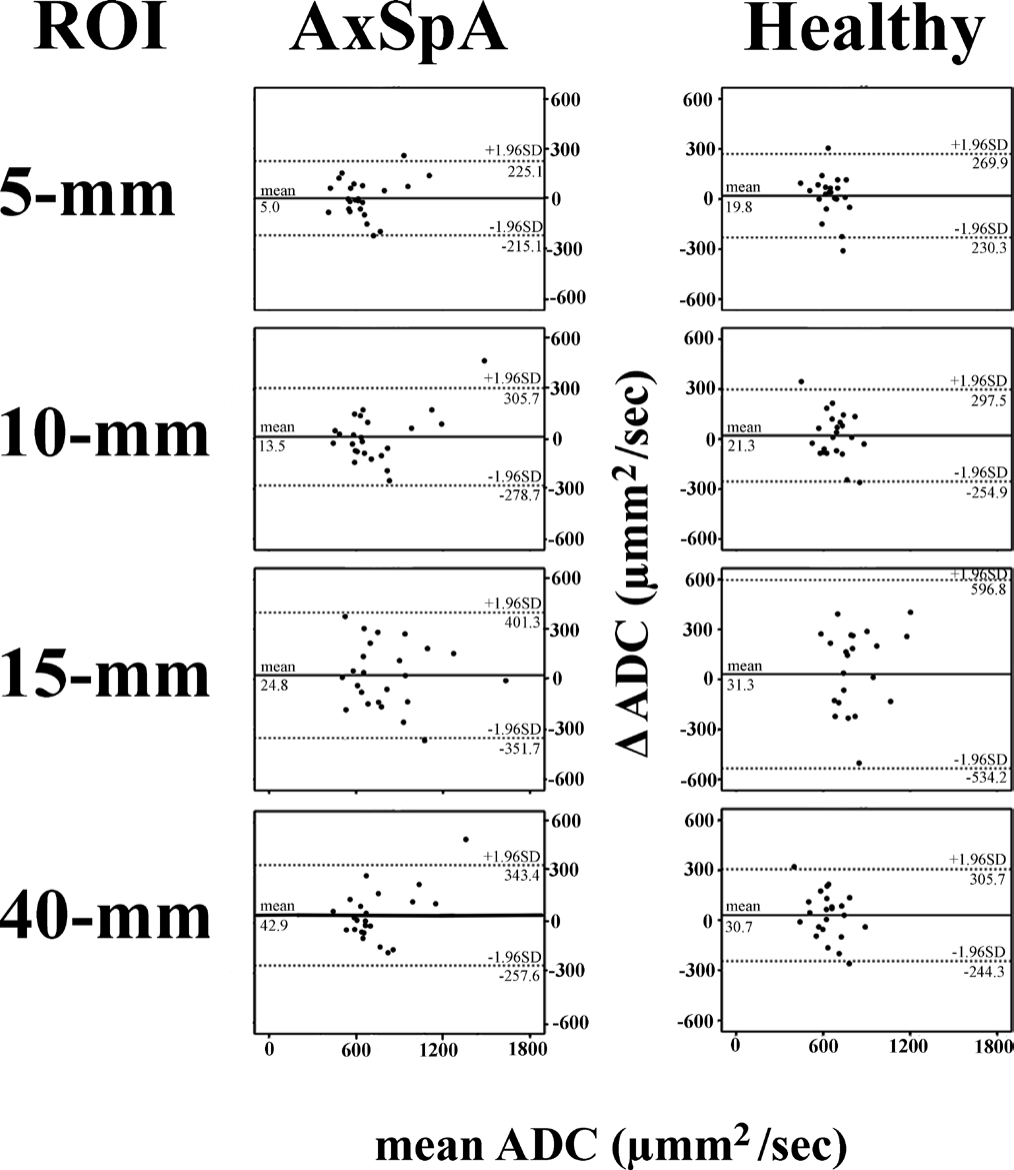
Bland–Altman plots of the median ADCs for the four different ROI settings. No systematic bias was found because for all four ROIs in both groups, the mean difference between the two MRI examinations was not significantly different from zero. The 95% limits of agreement (1.96 × SD) are shown as dotted lines. ADC, apparent diffusion coefficient; AxSpA, axial spondyloarthritis; ROI, region of interest; SD, Standard deviation.

**Figure 4.**
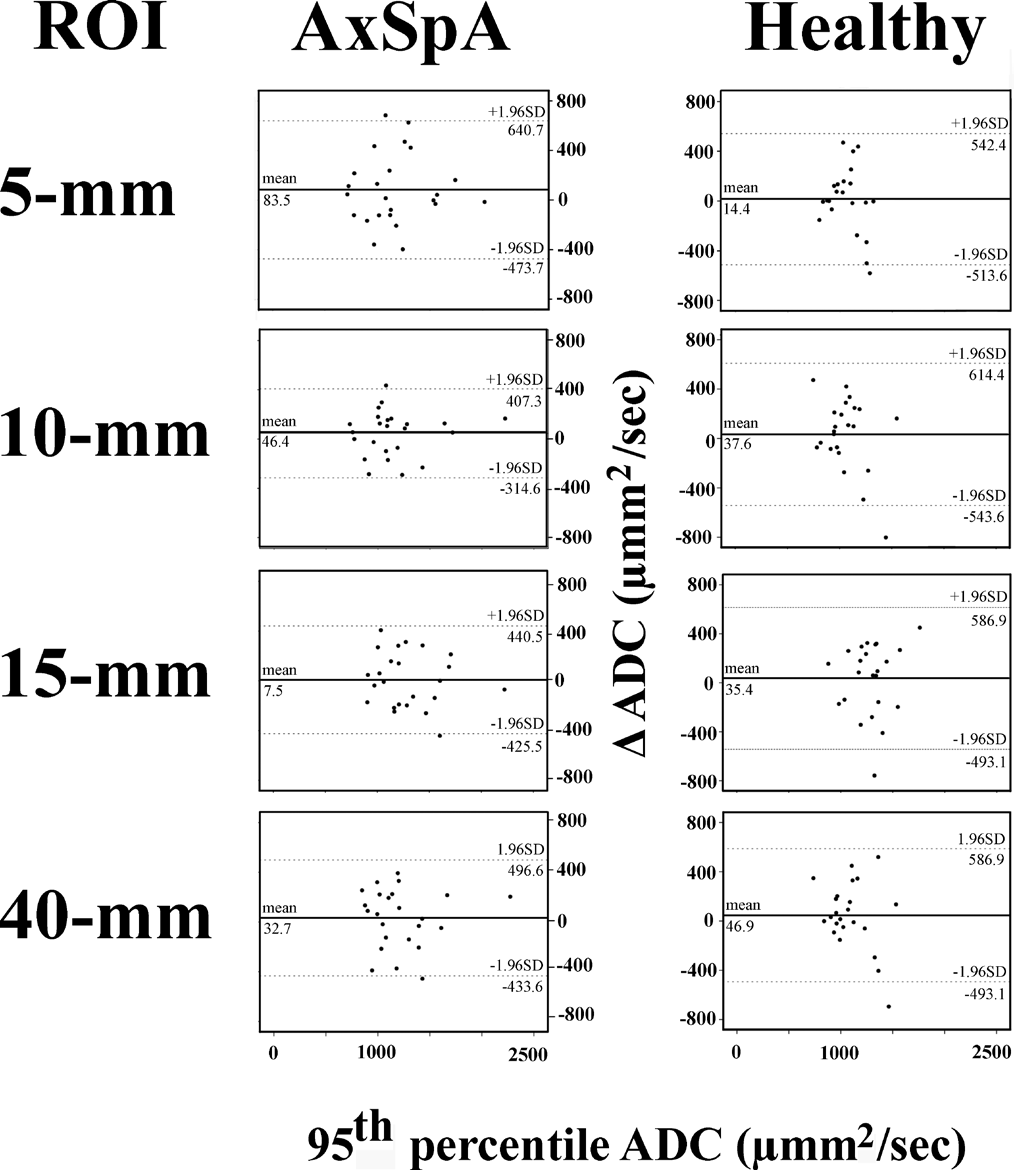
Bland–Altman plots of the 95th percentile ADCs for the four different ROI settings. Mean values are shown as solid lines and the 95% limits of agreement are shown as dotted lines. ADC, apparent diffusion coefficient; AxSpA, axial spondyloarthritis; ROI, region of interest.

Bland–Altman plots of ADC_med_ measurements (Figure 3) for the 5 mm band-shaped, 10 mm band-shaped, 15 mm linear, and 40 mm^2^ circular ROIs revealed small estimated biases of 19.8, 21.3, 31.3, and 30.7 µmm^2^ s^−1^ for the healthy subjects and 5.0, 13.5, 24.8, and 42.9 µmm^2^ s^−1^ for the patients with axSpA, respectively. The random errors were 127.6, 140.9, 288.5, and 140.3 µmm^2^ s^−1^ for the healthy subjects and 113.3, 149.1, 192.1, and 153.3 µmm^2^ s^−1^ for the patients with axSpA, respectively. Higher levels of estimated bias and random error were observed for the ADC_95_ measurements (Figure 4).

**Figure 5.**
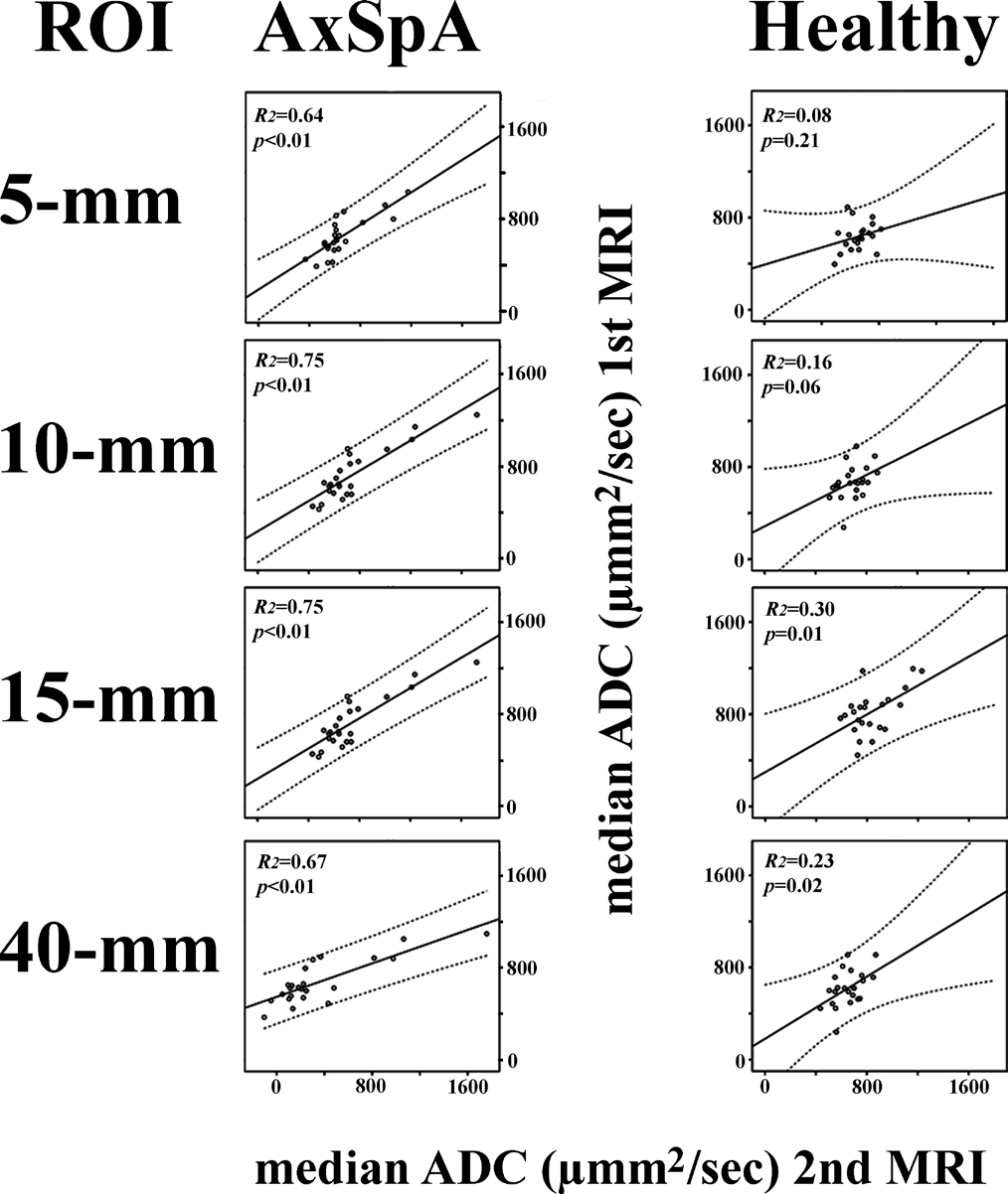
Scatter plots of the median ADCs at the first MRI *vs* the second MRI. Regression plots are shown as solid lines and 95% prediction limits are shown as dotted lines. The *p* and *R^2^* values are provided. ADC, apparent diffusion coefficient; AxSpA, axial spondyloarthritis; ROI, region of interest.

**Figure 6.**
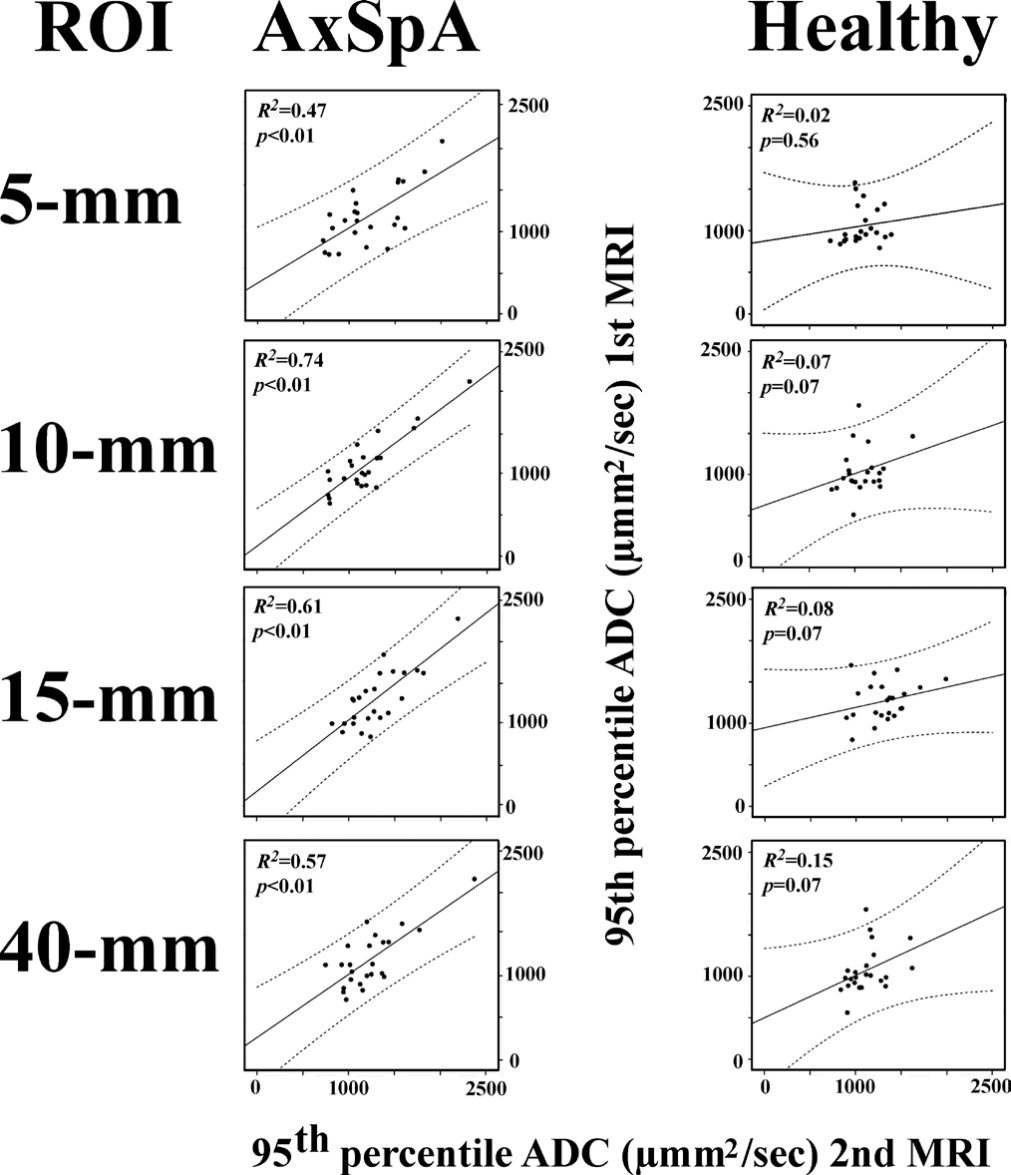
Scatter plots of the 95th percentile ADCs at the first MRI *vs* the second MRI. Regression plots are shown as solid lines and 95% prediction limits are shown as dotted lines. The *p* and *R^2^* values are provided. ADC, apparent diffusion coefficient; AxSpA, axial spondyloarthritis; ROI, region of interest.

Linear regression analyses of ADC_med_ measurements revealed a significant association between MRI1 and MRI2 examinations for all ROI settings in patients with axSpA and for the 15 mm linear and 40 mm^2^ circular ROIs, but not the 5 mm band-shaped and 10 mm band-shaped ROIs, in healthy subjects (Figure 5). Linear regression analyses of ADC_95_ measurements revealed significant associations for all ROI settings in patients with axSpA but not for any ROI settings in healthy subjects (Figure 6).

Table 1 shows the results of the interstudy repeatability and intrareader reproducibility analyses for the four different ROI settings. For all ROI settings, the interstudy repeatability of the ADC_med_ was good and ADC_95_ measurements was moderate to good in patients with axSpA and poor to fair in healthy subjects. For all ROI settings, the intrareader reproducibility of the ADC_med_ measurements was good to excellent and the intrareader reproducibility of the ADC_95_ measurements was fair to excellent. The interreader reproducibility was moderate for both ADC_med_ [ICC: 0.71 (95% CI (0.14–0.93)] and ADC95 [ICC: 0.68 (95% CI (0.08–0.92)]. The ADC_med_ measurement for the 15 mm linear ROI was significantly higher than those for the other ROI settings (5 mm band-shaped: *p* < 0.01; 10 mm band-shaped: *p* = 0.02; 40 mm^2^ circular: *p* < 0.01), whereas no significant differences were observed among the 40 mm^2^ circular, 5 mm band-shaped, and 10 mm band-shaped ROIs. The ADC_95_ measurement for the 15 mm linear ROI was significantly higher than those for the 5 mm and 10 mm band-shaped ROI settings (*p* = 0.02 and *p* = 0.01, respectively).

## Discussion

This study showed that there were no significant differences in interstudy repeatability (*i.e.* the repeatability between two subsequent sacroiliac joint MRI examinations) at four ROI settings, and that interstudy repeatability was markedly better in patients with axSpA than in healthy subjects. The estimated bias and random error were smallest for the 5 mm band-shaped ROI and the interreader reproducibility was moderately. The intrareader reproducibility was excellent in all subjects for most ROI settings. The ADC measurements were significantly higher for the 15 mm linear ROI than for the other ROIs. There were no significant changes in conventional MRI scores of SIJ inflammation and damage between the first and second MRI examinations.

To our knowledge, no other study has investigated the interstudy repeatability and intrastudy reproducibility of ADC measurements in SIJ bone marrow using several different ROI settings, but other studies that did not involve the bone marrow have been performed.^12–18^ Our approach was partly adapted from other methods. We measured predefined anatomy-based ROIs and did not restrict our approach to measuring lesion-based ROIs. Our circular ROI setting was adapted from those described in previous publications,^3,5,19,20^ our linear ROI setting was adapted from that described by Vendhal et al^21^, and our band-shaped ROI settings were customized for this study. Similar anatomy-based ROI settings have been used in studies on the liver, where the intrareader reproducibility was good (ICC, 0.75–0.81) but the interreader reproducibility was poor (ICC, 0.37–0.45).^14^ In a study of the parotid glands in patients with Sjögren’s syndrome, the intra- and inter reader reproducibility for three different ROI settings was good to excellent.^12^ As expected, these results are consistent with those of our study because anatomy-based ROIs are more likely to generate objective measurements than lesion-based ROIs. For each ROI setting, there are advantages and limitations. If only part of a joint is being assessed (*e.g.* a selected slice or a customized ROI), assessments can be completed more rapidly than if the whole joint is being evaluated. However, the particular ROI chosen may not adequately reflect bone marrow heterogeneity, and because different observers may choose slightly different ROIs, interreader variation may increase.^12^ Further, if ROIs are defined by the presence of a lesion (*e.g.* BME), it may be difficult to measure the response to treatment. This is because lesions can appear and disappear in different areas.^13^

The Bland–Altman plots revealed no systematic bias. Therefore, ADC measurements should be highly repeatable. The 5 mm band-shaped ROI showed the least estimated bias and random error in both healthy subjects and patients; therefore, we find that this ROI setting should be preferred. However, the linear regression analyses and the ICCs revealed that only ADC measurements from the patients with axSpA, and not those from the healthy subjects, were repeatable. The reason for this was a very small variance among the healthy subjects, but similar variance between the two MRI examinations as found for patients with axSpA. When calculating ICC, this resulted in decreased ICCs. Furthermore, because the ADC_med_ and ADC_95_ measurement ranges were small, the regression coefficients were low.^22^ Consequently, the ADC measurement confidence intervals are very wide for low and high values, making it difficult to compare them to those of patients. The ADC values for the 15 mm linear ROI setting were significantly different from those for the other three ROI settings. This is not surprising, because the 15 mm linear ROI setting included not only bone marrow but also cartilage and fluids inside the joint cavities. Similar results have been observed for rectal cancers,^16^ ovarian tumors,^15^ soft tissues,^17^ and anterior mediastinum,^18^ where whole volume (*e.g.* whole tumor) ADC measurements can differ from those from a predefined area.

By scoring the MRIs by the SPARCC method,^23,24^ which is the internationally most used method for scoring inflammation and damage in SIJ of patients with SpA, it was possible to assess if any changes in inflammation had occurred between the two MRI examinations. Because no significant changes were observed, all variations in the ADC measurements could be attributed to the scanner and the assessor.

Inflammation can be detected by use of several MRI modalities, where STIR displays BME,^23^ contrast-enhanced MRI reflects vascularity and leakage of contrast into the extravascular compartment,^25^ whereas DWI provides information on the velocity of movements of water molecules in the interstitial compartment between cells which among others depends on tissue cellularity.^25^ BME may be caused by other processes than inflammation and may persist for longer times, where DWI depends on tissue cellularity which potentially can change over shorter time periods because inflammatory cells have a short lifetime. In general radiologic practice, it can be challenging based on low-grade BME in SI joint to determine whether sacroiliitis is present or not^2^ and DWI may help to set the diagnosis as it may be superior to STIR in detecting inflammation.^26^ However, the age and sex dependency of ADC limits its use in discriminating inflamed lesions from degenerative lesions.^27,28^ Therefore, ADC seems to be more useable as an assessment tool, if the patient serves as its own control. Few studies have investigated this but both Bradbury et al^29^ and Bray et al^30^ have in small studies investigated therapy-induced changes using ADC, and they were able to monitor treatment with similar results as standardized BME scorings^29,30^ and Bray et al claimed ADC to be more objective than BME-scorings.^30^

The normal age-related conversion of red hematopoietic bone marrow to yellow fat-containing bone marrow results in changes in cellularity and hereby ADC,^25^ which limits the discrimination of uneven age groups. However, using normal-appeared bone marrow as reference an inflamed-to-normal bone marrow ratio can be calculated to overcome the age dependency.^30^

This study had some limitations. First, only one scanner in one hospital was used, and this was operated by the same two radiographers. In routine care, this situation would be different, decreasing the generalizability of our results. Moreover, several of our patients with axSpA did not have BME in the SIJs, which limits the generalizability of our results relative to those obtained by clinical trials in which patients may have BME in the SIJs due to study inclusion criteria.

In conclusion, the interstudy repeatability of systematic SIJ ADC measurements was independent of the ROI setting used. However, the 5 mm band-shaped ROI showed the least bias and random error and seemed preferable. The interstudy repeatability was high in patients with axSpA but not in healthy subjects. ADC measurements are affected by ROI settings, and this should be taken into account when assessing ADC maps.
